# Area postrema syndrome as the only sign of medullary infarction adjacent to area postrema

**DOI:** 10.1016/j.ensci.2025.100563

**Published:** 2025-03-14

**Authors:** Patrick Stancu, Nicolae Sanda, Karl-Olof Lovblad, Nils Guinand, Andreas Kleinschmidt, José Bernardo Escribano Paredes

**Affiliations:** a Department of Clinical Neurosciences, University Hospital, Geneva, Switzerland; b Department of Neuroradiology, University Hospital, Geneva, Switzerland; c Division of Otorhinolaryngology, University Hospital, Geneva, Switzerland

**Keywords:** Area postrema, Area postrema syndrome, Intractable nausea vomiting hiccups, Ischemic stroke, Neuromyelitis optica spectrum disorder

## Abstract

Area postrema syndrome (APS) is characterized by acute or subacute intractable nausea, vomiting, and/or hiccups lasting for at least 48 h. These symptoms can occur individually or in combination and are typically linked to periventricular brainstem lesions, particularly involving the area postrema (AP). The AP, a highly vascularized circumventricular organ located in the dorsal medulla oblongata, is supplied by the anterior spinal artery and perforating branches of the posterior inferior cerebellar artery (PICA), making it susceptible to pathological processes that can lead to APS. APS rarely occurs in stroke patients, but has been seen with ischemic lesions in the medial brachium pontis. The underlying pathophysiology of APS remains unclear, but remote lesions from the AP suggest involvement of an autonomic network of neuronal structures. This article reports a rare case of APS caused by ischemic stroke near the area postrema, without accompanying neurological impairments. The case highlights the importance of vascular investigation in intractable APS cases, even without focal neurological symptoms, and supports the role of neuronal structures connected to the AP in APS development.

## Highlights

• APS is defined by intractable nausea, vomiting, and/or hiccups lasting ≥48h , often due to periventricular brainstem lesions.
• APS can result from ischemic lesions within or near the area postrema.
• While APS pathophysiology remains unclear, remote lesions suggest involvement of a broader autonomic network.
• Comprehensive vascular assessment is essential in unexplained intractable nausea, vomiting, and hiccups.

## 1 Case report

**Fig. 1:**
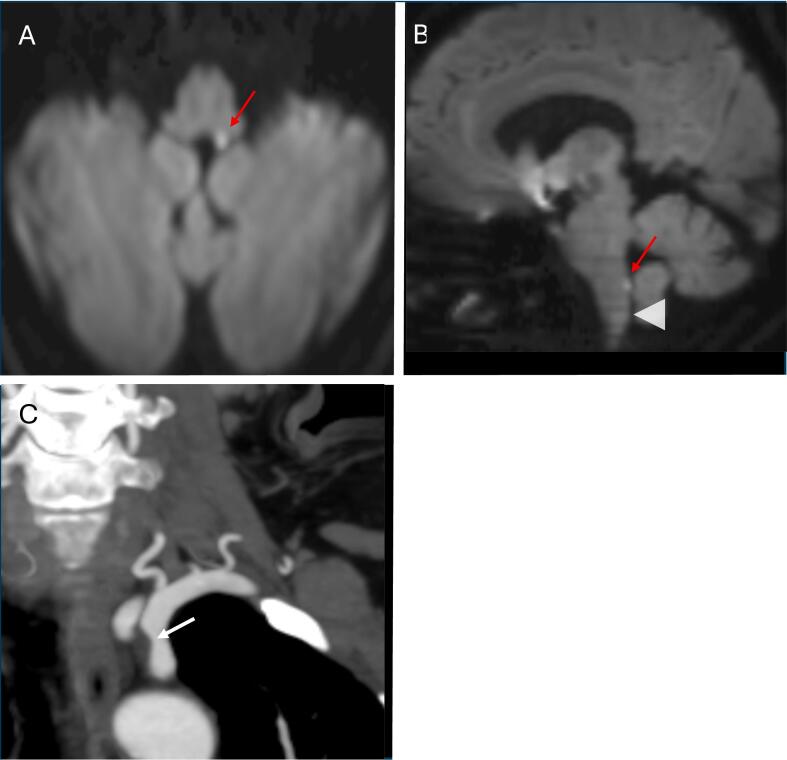
Brain MRI and CT angiography. Axial (A) and sagittal (B) diffusion-weighted imaging show a hyperintensity in the left posteromedial medulla, suggestive of an acute infarction. The topographic relationship between the ischemic lesion (red arrow) and the approximate location of the area postrema (arrowhead) in the dorsal medulla is demonstrated. Coronal (C) CT angiography reveals a non-significant (<50 %) stenosis (white arrow) of the proximal left subclavian artery due to a smooth plaque without calcification. (For interpretation of the references to colour in this figure legend, the reader is referred to the web version of this article.)

A 63-year-old woman with a history of well-controlled hypertension and diabetes presented with sudden-onset intractable nausea, vomiting, and hiccups following neck manipulation at the hairdresser. Later that same day, she experienced a spontaneous episode of mild dizziness, which resolved within one hour but prompted her to seek evaluation in the emergency department. On examination, her neurological assessment was unremarkable, and laboratory tests were within normal limits. Vestibular function analysis, including video-nystagmography, video head impulse testing, and vestibular-evoked myogenic potentials, revealed no abnormalities. Considering the patient's acute symptom onset, coupled with a transient episode of dizziness and the absence of other associated systemic signs, the neurology team was consulted and recommended excluding a cerebrovascular accident. Brain MRI (Fig. 1A–B) identified a punctiform ischemic lesion in the posteromedial medulla, located a few millimeters above the left area postrema (AP). The diagnostic work-up also identified a soft atherosclerotic plaque in the left subclavian artery (Fig. 1C), suggestive of an atheroembolic origin. Further etiological work-up, including cardiac and serological examinations, coagulation studies, blood and cerebrospinal fluid analyses, and anti-aquaporin-4 antibody testing, yielded negative results.

The patient was treated effectively with oral ondansetron as an antiemetic. Nausea and vomiting resolved within two days, while hiccups, though less frequent, subsided after one week. Following full recovery, she was discharged on day nine with aspirin and statin therapy.

## 2 Discussion

Area postrema syndrome (APS) is defined by acute or subacute intractable nausea, vomiting, or hiccups persisting for at least 48 h, occurring either in isolation or combination [1]. Although relatively rare, APS is most frequently reported in association with demyelinating lesions, particularly in neuromyelitis optica spectrum disorders (NMOSD), where it is recognized as a core clinical feature [1,2]. In contrast, APS secondary to ischemic stroke is uncommon [3]. However, a broad range of conditions can present with intractable nausea, vomiting, and hiccups (Table 1).

**Table 1:** Differential diagnosis of nausea, vomiting and intractable hiccups, adapted from [10].

CNS disorders	NMOSD, lateral medullary strokes, multiple sclerosis, MOG-Antibody Associated Disease, neurosarcoidosis, brainstem neoplasms and vascular malformations, Parkinson's disease, autoimmune GFAP astrocytopathy, LGI1-antibody encephalitis, neurosyphilis
Systemic autoimmune diseases	Systemic lupus erythematosus, giant cell arteritis
Cardiovascular/respiratory disorders	Aortic aneurysm, myocardial infarction, pericarditis and mediastinitis, asthma, bronchitis, diaphragmatic tumor or a hernia, empyema, pneumonia, pulmonary embolus
Gastrointestinal/ renal disorders	Bowel obstruction, esophageal neoplasm, esophagitis, gallbladder disease, gastritis, pancreatitis, peptic ulcer disease, renal abscess
ENT disorders	Laryngitis, neck cyst, pharyngitis, recent intubation
Infectious disorders	*Helicobacter pylori*, herpesviruses, influenza, malaria, tuberculosis
Metabolic/endocrine disorders	Hypocapnia, electrolyte imbalance, diabetes mellitus, thyrotoxicosis
Drugs	Dopamine agonists, aripiprazole, azithromycin, benzodiazepines, chemotherapeutics (ex. platinum-based therapy), dexamethasone, donepezil, ethanol, opioids (morphine, tramadol), anesthetic agents

CNS = Central Nervous System, NMOSD = neuromyelitis optica spectrum disorders, MOG = Myelin oligodendrocyte glycoprotein, GFAP = Glial Fibrillary Acid Protein, LGI1 = Leucine-rich glioma-inactivated 1, ENT = ear, nose and throat.

APS arises from lesions in the area postrema (AP) but can also result from more extensive periventricular brainstem involvement, affecting neighboring structures such as the nucleus tractus solitarius (NTS) and the ventrolateral respiratory center [3,4]. As these regions may constitute a neural network, a single lesion in any of them could independently lead to APS [5]. The frequent presence of nausea and vomiting in the posterior circulation infarctions, estimated to occur in approximately one-third of cases, is attributed to vestibular nucleus involvement [6]. While vomiting is common in strokes, particularly in large strokes and those affecting the posterior circulation, intractable hiccups are considerably less frequent [3,6]. Their co-occurrence with nausea and vomiting suggests involvement of the AP. However, when hiccups are absent, APS may not be identified, leading to underdiagnosis, particularly in lateral medullary [3]. Current understanding suggests that the neurocircuitry responsible for emesis involves multiple structures within the medullary reticular formation of the hindbrain, including the AP, NTS, dorsal motor nucleus of the vagus, reticular formation, and ventrolateral medulla [7]. While the precise neural network implicated in APS remains incompletely understood, the AP is widely recognized as a pivotal “vomiting center” [8]. It integrates chemical and neural inputs from the blood and brainstem, projecting to brain structures involved in autonomic control [7,9]. Damage to this region results in intractable nausea, emesis, and hiccups, leading to the clinical syndrome known as APS. Noteworthy, one case of stroke-related, isolated APS reported a small periventricular lesion remotely located near the brachium pontis, suggesting that damage to a broader autonomic control network may cause APS [5].

Our case of APS associated with a lesion located several millimeters superior to the AP supports this hypothesis. However, even though the primary region in the central nervous system associated with APS is the brainstem, Itabashi et al. reported cases of patients presenting with hiccups due to supratentorial infarcts in regions such as the insular cortex and temporal lobe [8]. Notably, in these cases, the hiccups were accompanied by other focal neurological signs. Although APS can be diagnosed based on a single symptom, such as persistent hiccups, the absence of brainstem involvement suggests that these cases should not be classified as APS [1,2]. APS is a rare clinical presentation of stroke, yet the presence of sudden-onset intractable nausea, vomiting, and hiccups should not be trivialized and must prompt comprehensive vascular investigations.

## Study funding

No targeted funding reported.

## Informed consent

The subject gave its informed consent for inclusion before participating in the study.

The patient has provided written consent for the publication of their imaging and medical history.

## Ethics approval

The study was conducted in accordance with the Declaration of Helsinki.

## CRediT authorship contribution statement

**Patrick Stancu:** Writing – original draft, Investigation, Conceptualization. **Nicolae Sanda:** Visualization, Validation, Supervision. **Karl-Olof Lovblad:** Writing – review & editing, Visualization. **Nils Guinand:** Validation, Supervision. **Andreas Kleinschmidt:** Writing – review & editing, Visualization, Validation. **José Bernardo Escribano Paredes:** Visualization, Validation, Supervision, Conceptualization.

## Declaration of competing interest

The authors report no disclosures relevant to the manuscript.
